# Expression of Nucleophosmin/NPM1 correlates with migration and invasiveness of colon cancer cells

**DOI:** 10.1186/1423-0127-19-53

**Published:** 2012-05-25

**Authors:** Yan Liu, Fei Zhang, Xiao-fang Zhang, Li-sha Qi, Lei Yang, Hua Guo, Ning Zhang

**Affiliations:** 1 Tianjin Medical University, Cancer Institute and Hospital, Research Center of Basic Medical Sciences, Key Laboratory of Breast Cancer Prevention and Therapy; Ministry of Education, Tianjin, 300060, China

**Keywords:** Human colon cancer, Nucleophosmin (NPM1), Invasion, Migration

## Abstract

****Background**:**

We aimed to examine the expression level of Nucleophosmin (NPM1) protein in colon cancer tissues and to investigate the potential role of NPM1 in the regulation of cell migration and invasiveness.

****Methods**:**

Immunohistochemical assay was performed to examine the expression pattern of NPM1 in 31 groups of colonic carcinoma samples, including colon tumors, adjacent normal tissues, and matched metastatic lymph nodes from the same patients. Small interfering RNA technique and exogenous expression of wild type NPM1 methods were used to further verify the function of NPM1.

****Results**:**

High-expression of NPM1 correlates with lymph node metastasis (*P* = 0.0003) and poor survival rate of human colon cancer patients (*P* = 0.017). SiRNA-mediated reduction of NPM1 was also shown to inhibit the migration and invasiveness of metastatic colon cancer HCT116 cell line. In addition, the exogenous expression of NPM1 in HT29 cells, a NPM1 low expression and low invasive colon cancer cell line, enhanced cell migration and invasiveness along with increased cell proliferation.

****Conclusions**:**

The current study uncovered the critical role of NPM1 in the regulation of colon cancer cells migration and invasion, and NPM1 may serve as a potential marker for the prognosis of colon cancer patients.

## **Background**

The incidence of colon cancer in China has increased in recent years and has become one of the most common malignancies, particularly in more developed areas [1]. Surgery and chemotherapy are highly effective for early-stage colon cancer [2]. However, the five-year survival rate of advanced colon cancer patients was very poor because of insensitivity to chemotherapy and more easily prone to recurrence [3]. Thus, the majority of patients with advanced-stage colon cancer die of metastasis. Molecular-targeted therapy has developed recently as a more effective approach for the treatment of metastatic colon cancer patients. However, whether the patients could receive the targeted drug therapy depends on the molecular profile of their cancer tissues. Until now, only a few effective targeted drugs are available for targeted therapy, such as bevacizumab, which binds to VEGF and inhibits the initiation of new blood vessel growth [4], and cetuximab, which targets EGFR and prevents the activation of cell growth signaling [5]. Screening and understanding novel genes involved in carcinogenesis or cancer metastasis is urgently needed to identify putative molecular targets for cancer therapy.

NPM1 is also called nucleophosmin, numatrin [6] or NO38 [7]. It consists of 294 amino acids. NPM1 is an abundant multifunctional nucleolar phosphoprotein, which shuttles constantly between the nucleus and cytoplasm and is involved in several important biological activities [8]. Previous studies demonstrated a critical role of NPM1 in ribosome biogenesis by regulating ribosome assembly and transporting ribosomal proteins to the cytoplasm. NPM1 also plays an essential role in cell growth and proliferation by regulating cell cycle progression and centrosome duplication [9,10]. Other studies also showed that NPM1 could regulate the activity of several tumor suppressors, including P53 and Rb by direct binding [11]. NPM1 is also involved in transcription activation through interaction with transcription factors, such as c-Myc and NF-κB [12]. In addition, NPM1 also functions as a molecular chaperone to regulate DNA histone assembly and proper folding of unfolding proteins [13].

Numerous studies revealed a critical role of NPM1 in tumorigenesis [14]. Altered NPM1 expression was observed in many types of tumors, and mutated NPM1 is frequently detected in human hematopoietic malignancies, especially in acute myeloid leukemia (AML) [15]. Recent research demonstrated that NPM1 may be a favorable prognosis marker for AML and even a potential therapeutic target in hematological malignances [16]. In contrast to AML, mutations of NPM1 are rarely detected in solid tumors. However, over-expression of NPM1 is often found in many kinds of solid tumors [17,18]. Accumulative evidence suggested that highly expressed NPM1 correlated with the stage of tumor progression and poor prognosis [19]. Although the function of NPM1 in carcinogenesis has been intensively studied, the detailed mechanism is largely unknown.

In the current study, the expression pattern of NPM1 in colon cancer tissues and adjacent normal tissues was examined. Increased expression of NPM1 was also found to be associated with lymph node metastasis and poor survival of colon cancer patients. The current study hypothesized that NPM1 may play a role in cancer cell migration and invasion. Small interfering RNA (siRNA) induced the down-regulation of NPM1-inhibited migration and invasiveness of metastatic colon cancer in the HCT116 cell line. Furthermore, exogenous expression of wild-type NPM1 in HT29 cells enhanced cell migration and invasiveness along with increased cell proliferation. These results revealed that NPM1 serves as an important regulator for cancer cell invasion.

## Methods

### Cancer tissues and immunohistochemistry

Colon cancer tissues, adjacent normal tissues, and matched metastatic lymph nodes were obtained from patients diagnosed with colon cancer in Tianjin Medical University Cancer Institute and Hospital (Tianjin, P. R. China) between 2000 and 2005. Immunohistochemical assay was performed as described previously [20]. In brief, tissue slides were deparaffinized in xylene and rehydrated with graded ethanol. The slides were heat induced in sodium citrate buffer for antigen retrieval, blocked in 3% BSA for 40 min, and incubated with mouse anti-human NPM1 antibodies (Santa Cruz Biotechnology, CA, USA) (1:50) at 4°C overnight. Detection was performed using an HRP-linked secondary antibody and a DAB kit. The slides were then counterstained with hematoxylin and mounted. Highly expressed NPM1 in human breast cancer tissue was used as a positive control. A negative control was prepared by omitting the primary antibody. The staining intensity and percentage of positive cells were scored semi-quantitatively by two pathology doctors. The intensity of staining was classified into four categories: "0" for no brown particle staining, "1" for light brown particles, "2" for moderate brown particles, and "3" for dark brown particles. The percentage of positive cells was divided into four groups: "0" for <10% positive cells, "1" for 10%–40% positive cells, "2" for 40%–70% positive cells, and "3" for ≥70% positive cells. The sum of the intensity and percentage of positive staining was used to determine high (score ≥ 3) or low (score < 3) expression of NPM1.

### Cell culture

Human colon cancer cell lines HCT116 and HT29 was purchased from Cell Resource Center, Institute of Basic Medical Sciences, Chinese Academy of Medical Sciences/Peking Union Medical College (Beijing, China). HCT116 cells were maintained in Iscove’s Modified Dulbecco’s medium (IMDM) (Hyclone, USA) supplemented with 10% fetal bovine serum (FBS, Hyclone, USA), HT29, SW480, Colo205 and SW620 cells were cultured in Dulbecco's Modified Eagle Medium supplemented with 10% FBS and 100U of penicillin and streptomycin (Hyclone, USA).

### Plasmid construction, siRNA and plasmid transfection

NPM1 specific siRNA (Sense: 5' -CCUAGUUCUGUAGAAGACAtt-3' and Antisense: 5' -ttUGUCUUCUACAGAACUAGGsiRNA-3') was synthesized by Genechem Co (Shanghai, P. R. China). Full length NPM1 was amplified by PCR with the followed primers: Forward: 5'-GCGCTCGAGATGGAAGATTCGATGGACATG-3'reverse: 5'-CGCGAATTCAAGAGACTTCCTCCACTG-3'. The PCR products were purified, digested and cloned into pEGFP-C3 vector (BD biosciences Clontech, CA) in the EcoR I and Xho I site (NEB, USA). The construct was verified by enzyme digestion and DNA sequencing. For transfection, human colon cancer cell lines HCT116 and HT29 were cultured to 60% -70% confluence in 6-well plates, then 100 pmol siRNA duplexes or 4 μg NPM1 expression plasmids and control vector were transfected with Lipofectamine 2000 (Invitrogen, Carlsbad, CA) according to the manufacturer’s instructions. After transfection for 48 hours, cells were harvested and used for further assays.

### Cell migration and invasion assays

Cell migration and invasion assays were performed as described previously [21]. Briefly, 48 hours after transfection, control and NPM1 knockdown cells or NPM1 over-expression cells were harvested, washed and resuspended, then 5 × 10^5^ cells in 400 μl serum free medium was added into the transwell inserts with 8μm pore polycarbonate filters which coated with (invasion) or without matrigel (migration) (BD Biosciences, San Jose, CA). After 24h (migration) or 48h (invasion) incubation, non-migrated cells on the top of the membrane were removed with a cotton swab. Cells migrated to the bottom sides of the membrane were fixed and stained. The number of migrated cells on the membrane was then counted in 3 randomly selected fields and taken the picture under a light microscope (Nikon SMZ 1000) at 400× magnification. All the experiments were performed at least three times.

### MTT assay

Control and siNPM1 colon cancer cells or NPM1 over-expression cells were plated in a 96-well microplate at densities of 2000 per well. The cells were cultured continuously for 4 days, upon analysis, 20μl of MTT reagent (5 mg/ml, Sigma, USA) was added into each well and the cells were incubated at 37°C for 4 h. Then the supernatant was discarded and 150ul of DMSO was added into each well, after incubation at 37°C for 30min, the absorbance was measured at 570 nm.

### BrdU incorporation assay

Cell proliferation assay was performed using a Cell Proliferation ELISA System (Roche Molecular Biochemicals, Mannheim, Germany) according to the manufacturer’s protocol. Cells were seeded onto 96-well plates at a density of 5000 cells per well and cultured for 24 h. BrdU solution (10 μM) was added to each well and the cells were incubated for another 4 h. The cells were then fixed, denatured, and immunostained with anti-BrdU antibody. BrdU incorporation was measured using an ELISA plate reader at 450 nm absorbance.

### Immunocytochemistry

Colon cancer cells were grown on sterile slips for 24 h, washed three times with PBS, and fixed in ice-cold ethanol for 10 min. Endogenous peroxidase activity was suppressed by incubation with 3% H_2_O_2_ in methanol for 10 min. After blocking with normal serum for 30 min, the cells were incubated with a working solution containing mouse anti-human proliferating cell nuclear antigen (PCNA) antibodies (ZhongShan Co., China) for 2 h at room temperature. Incubation with HRP-conjugated secondary antibody for 1 h at 37°C followed. The signal was detected using a commercial kit (Dako EnVision System, CA, and USA). The slides were mounted and observed under a light microscope (Nikon SMZ 1000) at 400× magnification. Five areas were randomly chosen to count the number of PCNA-positive staining cells. The PCNA staining index was calculated by counting the number of PCNA-positive staining cells per 100 cells.

### Wound healing assay

Cells were plated in 35 mm dishes at a density of 7.5 × 10^5^ cells per ml and cultured to form confluent cell monolayers. Then cells were scraped with a 10 μl pipette tip and washed with PBS to remove nonadherent cells, followed by incubation in medium containing 0.5% FBS at 37°C for 0, 3, 6, 9, 12 and 24 h. The wound areas were calculated and photographed under a phase contrast microscopy at 100× magnifications (Olympus CKX41, JP). The migration distance was quantified by subtracting the width of the wounds at each time point from the width of the initial wounds. All experiments were repeated three times, and the quantitative data were expressed as mean ± SD.

### Western blot analysis

Control group and experimental group of cells were plated on 10cm dish and cultured to 90% confluence. The cells were washed three times with cold PBS and lysed in 1x SDS cell lysis buffer containing a protease inhibitor cocktail (Roche Applied Science, Indianapolis, IN). Then, the cell lysates were boiled at 95°C for 5 minutes, cooled on ice, after centrifugation at 12,000g at 4°C for 15 minutes, protein concentration was determined by using a BCA™ Protein Assay Kit (Thermo, USA). 15ug of lysates was separated by 10% SDS-PAGE and transferred onto a PVDF membrane (Millipore, Bedford, MA), then the membrane was blocked with 5% nonfat milk for 1 h and incubated with monoclonal mouse anti-human NPM1 antibody (Santa Cruz, Biotechnology, CA 1:2000) overnight at 4°C. The membrane was then washed three times with TBST (TBS + 0.1% Tween-20) and incubated with a HRP-conjugated secondary antibody for 1 hour at room temperature, β-actin (Santa Cruz, Biotechnology, CA) (1:2500) was used as a loading control. An enhanced chemiluminescence reagents ECL kit (Sigma, USA) was used to examine the expression of indicated proteins.

### Statistical analysis

All data were analyzed by Statistical Package for the Social Sciences (SPSS 17.0 Chicago, IL) and GraphPad Prism 5 statistical software (San Diego, CA, USA). Chi-squre test or student’s T-test (two-tailed) and one-way ANOVA were used for data analysis. The survival curve of patients was calculated by Kaplan-Meier method. A *P* value less than 0.05 was considered statistically significant.

## Results

### High-expression of NPM1 is associated with lymph node metastasis in colonic carcinoma

**Figure 1:**
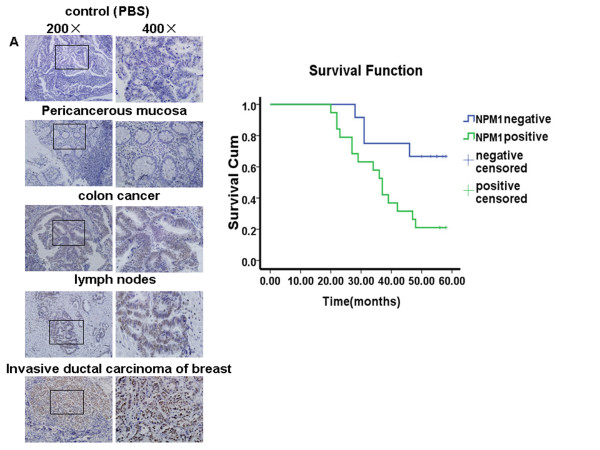
**Increased NPM1 expression was associated with lymph node metastasis and poor survival rate of colon cancer patients.A.** Immunohistochemical analysis of NPM1 expression in colon cancer tissues, adjacent normal tissues, and matched metastatic lymph nodes from the same patients. A primary antibody replaced with PBS was used as a negative control, and breast cancer tissue known to express high levels of NPM1 was used as a positive control. **B.** Kaplan–Meier estimates of the overall survival rate according to the expression of NPM1 (*P* = 0.017).

The expression level of NPM1 in 31 groups of colonic carcinoma tissues, including colon tumors, adjacent normal tissues, and matched metastatic lymph nodes from the same patients was investigated. Positive staining was observed in 19 cases of cancer tissues and 23 cases of metastatic lymph nodes. However, positive staining was observed in only eight cancer-adjacent normal tissues (Figure. 1A, Table 1). Statistical analysis showed a high expression of NPM1 correlated with lymph node metastasis (*P* = 0.0003), indicating an association between NPM1 in tumorigenesis and cancer metastasis. Among 13 cases of cancer tissues with distant metastasis, 12 showed positive staining for NPM1. Among 18 cases of cancer tissues without distant metastasis, only 7 exhibited positive staining (*P* = 0.003). These data suggested that the high expression of NPM1 in patients was correlated with increased distant metastasis. A statistically significant correlation was also observed between high NPM1 expression and poor survival rate compared with low NPM1 expression (*P* = 0.017) (Figure. 1B, Table 2). Thus, high NPM1 expression may be a poor prognosis marker for colon cancer patients.

**Table 1:** Expression of NPM1 in colon cancer tissues, adjacent normal tissues and matched metastatic lymph nodes

		**NPM1 expression**	
	**Total**	**Positive**	**Negative**
**Adjacent normal tissues**	**31**	**8**	**23**
**Colon cancer tissues**	**31**	19*	**12**
**Metastatic lymph nodes**	**31**	**23**^**#**^	**8**

Compared with adjacent normal tissues P = 0.0098; # compared with adjacent normal tissues P = 0.0003.

**Table 2:** Correlation of NPM1 expression with clinicopathologic parameters of colon cancer patients

		**NPM1 expression**		
**Parameters/Markers**	**Total**	**Negative**	**Positive**	***P*-value**
**Age (year)**				
**<40**	**4**	**1**	**3**	**0.813**
**40-60**	**19**	**8**	**11**	
**>60**	**8**	**3**	**5**	
**Sex**				
**Male**	**13**	**8**	**5**	**0.060**
**Female**	**18**	**4**	**14**	
**Size(cm)**				
**<4**	**10**	**5**	**5**	**0.308**
**≥4**	**21**	**7**	**14**	
**Position**				
**Ascending colon**	**17**	**5**	**12**	**0.272**
**Transverse colon**	**2**	**1**	**1**	
**Descending colon**	**2**	**0**	**2**	
**Sigmoid colon**	**10**	**6**	**4**	
**Distant metastasis**				
**Negative**	**18**	**11**	**7**	0.003*
**Positive**	**13**	**1**	**12**	

*P **< 0.05.**

### Expression of NPM1 was elevated in highly invasively colon cancer cell line

**Figure 2:**
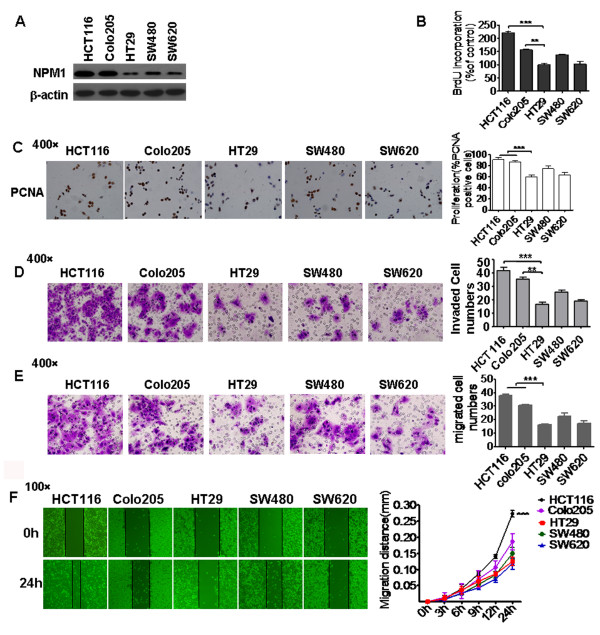
**Expression of NPM1 was elevated in highly invasive colon cancer cell line HCT116.A.** Western blotting analysis of NPM1 expression in five different colon cancer cell lines with distinct invasive potentials; β-actin was used as a loading control. **B.** Cell proliferation was measured by the BrdU incorporation assay. The proliferation rates of HCT116 and Colo205 were significantly higher than those of HT29. ***P < 0.0001, **P < 0.001. **C.** Immunocytochemical staining of PCNA in different cell lines. Expression of PCNA was higher in the HCT116 and Colo205 cell lines than in the HT29 cell line. Picture was taken with a light microscope at 400 magnifications. ***P < 0.0001. **D.** HCT116 and Colo205 cells exhibited higher invasive ability than HT29 cells. 5 × 10^5^ cells in 400 ul serum free medium were loaded into transwell inserts with 8μm pore polycarbonate filters which coated with matrigel. After 48h incubation, the invaded cells on the bottom side of the membrane were fixed and stained with 0.005% crystal violet. Then,the number of invaded cells was counted in three randomly chosen areas (400 × magnification) and summarized in the right panel. ***P < 0.0001, **P < 0.001. **E.** HCT116 and Colo205 cells exhibited higher migration ability than HT29 cells. Migration assay was performed the same as transwell invasion assay except that there was no coating matrigel on the filters. ***P < 0.0001. **F.** Representative images of wound healing assays at 0 and 24 h (100 × migration). The migration distance was quantified by subtracting the width of the wounds at each time point from the width of the initial wounds. HCT116 cells showed increased migration ability compared with the other cell lines. ***P < 0.0001.

To investigate the relationship between NPM1 and cancer cell aggressive, the expression level of NPM1 in five different colon cancer cell lines with distinct invasive potential was examined using the Western blotting method. NPM1 was found to be strongly expressed in the HCT116 cell line, but weakly in the HCT29 cell line, a low metastatic colon cell line [22-26] (Figure 2A). We also examined the invasion and migration potential of five colon cancer cell lines by transwell and the wound healing assays. As shown in Figures 2D and 2E, transwell assays showed that HCT116 cells exhibited higher invasion and migration ability than HT29 cells; similarly, wound healing assay also showed that directional migration of HCT116 cells was higher than HT29 cells (Figure 2F). These data were consistent with previous reports that HCT116 is a highly aggressive cell line [24,27]. Therefore, NPM1 may play a role in the enhanced invasiveness of colon cancer cells.

### Down-regulation of NPM1 inhibited cancer cell proliferation

**Figure 3:**
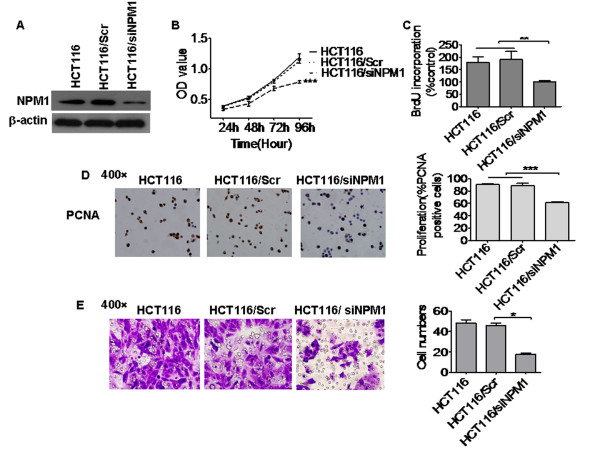
**Knock down of NPM1 expression inhibited human colon cancer cell migration, invasion and proliferation. A.** Western blotting analysis of NPM1 expression in HCT116 cells, HCT116 cells transfected with NPM1 specific siRNA and a control siRNA (Scr). β-actin was used as a loading control. **B.** MTT assay showed that the knockdown of NPM1 expression inhibited cell proliferation. Data were presented as mean ± SD. ***P < 0.0001. **C.** BrdU incorporation rate decreased after the knockdown of NPM1 in HCT116 colon cancer cells. **P < 0.001. **D**. PCNA expression in the nucleus was reduced after the down-regulation of NPM1. The PCNA staining index was calculated by counting the number of PCNA-positive staining cells per 100 cells in five randomly chosen areas. ***P < 0.0001. **E.** Knockdown of NPM1 decreased colon cancer cell invasion. Cell suspension with serum-free medium was loaded into the matrigel-coated upper chamber, and FBS-containing media were loaded into the lower chamber. The invaded cells were counted in three randomly chosen areas. *P < 0.05.

Previous studies have demonstrated that NPM1 is overexpressed in several solid tumors, and contributed to cell growth and proliferation [17,18,28,29]. The current study investigated the different proliferation rate of five colon cancer cell lines in native conditions using the MTT and BrdU incorporation methods. Cell line HCT116 with highly expressed NPM1 had a higher proliferation rate than other cells (Figures 2B and 2C). To validate further the relationship between NPM1 and cell proliferation, a siRNA sequence was used to knockdown the expression of NPM1 in colon cancer cells. Compared with the control siRNA, transient transfection with NPM1-specific siRNA successfully downregulated NPM1 expression in HCT116 cells (Figure 3A). The MTT and BrdU incorporation assay results showed that cells with NPM1 knockdown exhibited decreased cell proliferation (Figures 2B and 3C) and decreased PCNA expression (Figure 3D). This finding indicated that NPM1 function was inhibited in HCT116 cells, consistent with previous reports [30-32].

### Reduction of NPM1 impaired Human colon cancer cell migration and invasion

The current study speculated that NPM1 may play a role in the elevated invasive potential of cancer cells and investigated the effect of NPM1 knockdown on cancer cell migration and invasion using a Boyden chamber assay. The current data showed that the migration and invasiveness of HCT116 cells transfected with NPM1-specific siRNA significantly decreased compared with that of the cells transfected with control siRNA ( Additional file 1,Figure 3E ). These results suggested that NPM1 plays an important role in the migration and invasion of colon cancer cells.

### Exogenous expression of NPM1 enhanced migration and invasion ability of HT29 cells

**Figure 4:**
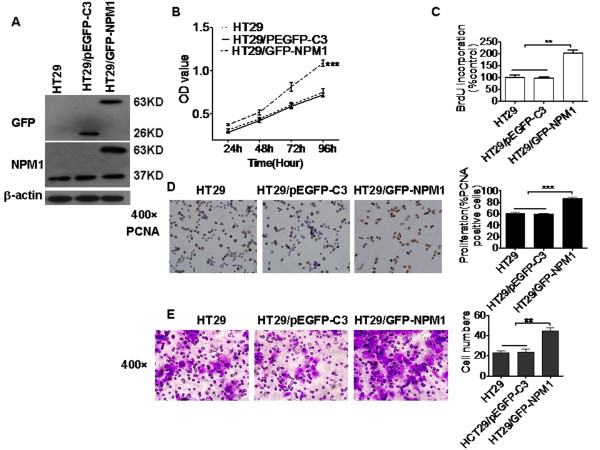
**Exogenous expression of NPM1 enhanced colon cancer cell migration and invasive ability. A.** Western blotting analysis of NPM1 expression in HT29 cells transfected with control or GFP-tagged NPM1 plasmid. NPM1 and GFP antibody were used to confirm the expression of NPM1. β-actin was used as a loading control. **B.** MTT assay showed that elevated NPM1 expression in human colon cancer HT29 cells increased cell proliferation. ***P < 0.0001. **C.** BrdU incorporation rate increased after exogenous NPM1 expression in HT29 cells. **P < 0.001. **D.** PCNA expression in the nucleus increased after exogenous NPM1 expression in HT29 cells. ***P < 0.0001. **E.** Elevated NPM1 expression in human colon cancer HT29 cells enhanced the cell invasive ability. **P < 0.001.

To further reveal the role of NPM1 in the regulation of cancer cell migration and invasion, EGFP tagged wild-type NPM1 construct were transfected into HT29 colon cells, which exhibited weak migration and invasiveness. Transfection of NPM1 construct in HT29 cells led to increased expression of NPM1, NPM1 expression in HT29 cells were also confirmed by Western blotting using GFP antibodies. As shown in (Figure 4A). Migration and invasion assay results showed that elevated NPM1 expression in HT29 cells enhanced cell migration and invasive ability, compared with that of the cells transfected with empty vectors (Additional file 2,Figure 4E). In addition, HT29 cells transfected with wild-type NPM1 were also found to display a significant increase in cell proliferation (Figures 4 B and 4C), meanwhile, PCNA expression in nucleus was also elevated (Figure 4D).

## Discussion

NPM1 is a multifunctional nucleolar phosphoprotein that plays an essential role in cell growth and proliferation by regulating cell cycle progression,ribosome biogenesis, and centrosome duplication [10,33,34]. NPM1 is also crucial for maintaining genomic stability by influencing DNA replication and repair [35]. The molecular chaperone activity of NPM1 is critical for DNA-histone assembly and proper protein folding. In addition, NPM1 is involved in transcription activation through its interaction with transcription factors, such as c-Myc, NF-κB, and FOXM1 [36-38]. Given the fact that NPM1 functions as a key regulator for many cellular activities [35], the contribution of NPM1 to oncogenesis has received much attention. Interestingly, previous studies revealed that NPM1 acts as both tumor suppressor and proto-oncogene during tumorigenesis [39]. On one hand, NPM1 functions as a tumor promoter through the inhibition of several tumor suppressors including P53 and Rb via direct binding. NPM1 can also interact with proto-oncogene c-Myc and enhance its transforming activity [11]. On the other hand, NPM1 is a potential tumor suppressor by maintaining chromosome stability and regulating ARF activity [40,41].

Numerous studies demonstrated that the contribution of NPM1 to tumorigenesis depend on its expression level and genetic modifications [42]. Most studies on NPM1 concentrated on human hematopoietic malignancies, and mutations of NPM1 are frequently observed in these tumors, especially in AML. In contrast to AML, a recent study found that mutated NPM1 is rarely detected in solid tumors. Instead, altered expression of NPM1 was observed in many types of solid tumors, and the detailed functions are just starting to be clarified [43]. In the current study, evidence that NPM1 expression was remarkably increased in the colon cancer tissues and metastatic lymph nodes compared with the adjacent noncancerous tissues was provided. Statistical analysis showed that the overexpression of NPM1 was associated with distant metastasis, suggesting that NPM1 might play a critical role in colon carcinogenesis and colon cancer metastasis. Another study also showed that increased NPM1 expression correlated with tumor progression in hepatocellular carcinoma [44], which was consistent with the current result. In addition, a positive correlation was also found between the high expression of NPM1 and the poor five-year survival performance of colon cancer patients. Interestingly, similar results were also observed in other solid tumors [45,46], indicating that NPM1 may be a potential prognosis marker for cancer patients.

Although much evidence demonstrates that NPM1 expression correlates with clinical parameters of cancer patients, the detailed role of NPM1 in cancer progression is largely unknown. The current finding on the increased expression of NPM1 in highly metastatic HCT116 colon cancer cells probably indicate that NPM1 is involved in colon cancer metastasis, in addition to cell growth regulation and proliferation. In support of this hypothesis, the present results showed that siRNA induced the down-regulation of NPM1-inhibited migration and invasion of human colon cancer cell HCT116 along with decreased cell proliferation. Furthermore, exogenous expression of wild-type NPM1 in HT29 cells enhanced cell migration and invasiveness. Migration and invasion is critical for cancer cell metastasis, thus, the current results provided direct evidence that the expression level of NPM1 is necessary for colon cancer cell metastasis. Interestingly, a recent study reported that mutant NPM1 promoted migration and invasion in NIH3T3 cells [20]. In contrast to this finding, mutations of NPM1 inhibited cell migration in HEK293 cells [47]. The effect of NPM1 on cell migration may be cell type-dependent because NPM1 mutations are a rare event in most solid tumors [46]. However, its detailed role in cancer cell metastasis needs further investigation.

## **Conclusion**

The current results suggested that the elevated expression of NPM1 correlated with distant metastasis and poor survival of colon cancer patients. The current study also provided the first direct evidence that the expression level of NPM1 is critical for colon cancer cell migration and invasion. Although the detailed mechanism of NPM1 in colon carcinogenesis remains to be elucidated, the present study speculated that NPM1 may be a potential prognosis biomarker and a possible therapeutic target for colon cancer patients.

## Abbreviations

NPM1: Nucleophosmin:; AML: acute myeloid leukemia; PCNA: proliferating cell nuclear antigen.

## Competing interests

The authors declare there is no conflict of interests

## Authors' contributions

YL, FZ, NZ, conceived the study, designed the experiments; YL performed the experiments and data analysis; LY collected clinical data. XFZ, LSQ, HG read and revised the final manuscript. All authors read and approved the final manuscript.

## Supplementary Material

Additional file 1 Knockdown of NPM1 decreased colon cancer cell migration. Cell migration assay was performed by using transwell insert without coating matrigel in the upper chamber; Cell suspension with serum free media was loaded into upper chamber, and FBS containing media were loaded into the lower chamber. Then invaded cells in three random chosen areas were counted. ** *P* < 0.001. Click here for file

Additional file 2 Elevated NPM1 expression in human colon cancer HT29 cells promotes cells migration ability. High expression of NPM1 enhanced migration ability of HT29 cells. * *P* < 0.05. Click here for file
