# Supplementary figures and images for: Expression of Nucleophosmin/NPM1 correlates with migration and invasiveness of colon cancer cells
Source: J Biomed Sci. 2012 May 25;19(1):53. doi: 10.1186/1423-0127-19-53 (PMC3404909; doi:10.1186/1423-0127-19-53)

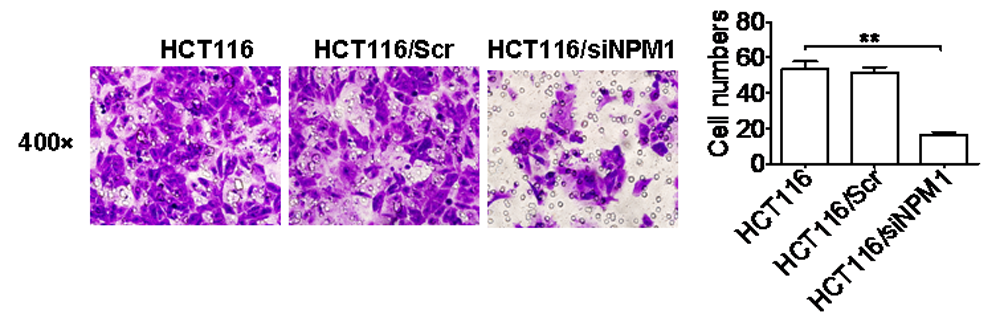

Supplement: Additional file 1 — Knockdown of NPM1 decreased colon cancer cell migration. Cell migration assay was performed by using transwell insert without coating matrigel in the upper chamber; Cell suspension with serum free media was loaded into upper chamber, and FBS containing media were loaded into the lower chamber. Then invaded cells in three random chosen areas were counted. ** P < 0.001. [file 1423-0127-19-53-S1.tiff]

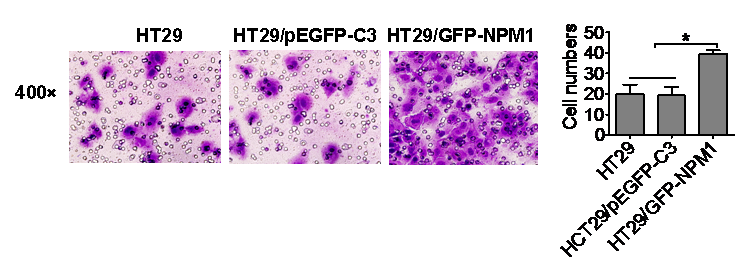

Supplement: Additional file 2 — Elevated NPM1 expression in human colon cancer HT29 cells promotes cells migration ability. High expression of NPM1 enhanced migration ability of HT29 cells. * P < 0.05. [file 1423-0127-19-53-S2.tiff]
